# Improved contrast of affibody-mediated imaging of HER3 expression in mouse xenograft model through co-injection of a trivalent affibody for *in vivo* blocking of hepatic uptake

**DOI:** 10.1038/s41598-019-43145-2

**Published:** 2019-05-01

**Authors:** Maria Rosestedt, Ken G. Andersson, Sara S. Rinne, Charles Dahlsson Leitao, Bogdan Mitran, Anzhelika Vorobyeva, Stefan Ståhl, John Löfblom, Vladimir Tolmachev, Anna Orlova

**Affiliations:** 1 Department of Medicinal Chemistry, Uppsala University, Uppsala, Sweden; 2 Department of Protein Science, KTH – Royal Institute of Technology, Stockholm, Sweden; 3 Department of Immunology, Genetics and Pathology, Uppsala University, Uppsala, Sweden; 4 Science for Life Laboratory, Uppsala University, Uppsala, Sweden

**Keywords:** Cancer imaging, Cancer imaging

## Abstract

Human epidermal growth factor receptor type 3 (HER3) plays a crucial role in the progression of many cancer types. *In vivo* radionuclide imaging could be a reliable method for repetitive detection of HER3-expression in tumors. The main challenge of HER3-imaging is the low expression in tumors together with endogenous receptor expression in normal tissues, particularly the liver. A HER3-targeting affibody molecule labeled with radiocobalt via a NOTA chelator [^57^Co]Co-NOTA-Z_08699_ has demonstrated the most favorable biodistribution profile with the lowest unspecific hepatic uptake and high activity uptake in tumors. We hypothesized that specific uptake of labeled affibody monomer might be selectively blocked in the liver but not in tumors by a co-injection of non-labeled corresponding trivalent affibody (Z_08699_)_3_. Biodistribution of [^57^Co]Co-NOTA-Z_08699_ and [^111^In]In-DOTA-(Z_08699_)_3_ was studied in BxPC-3 xenografted mice. [^57^Co]Co-NOTA-Z_08699_ was co-injected with unlabeled trivalent affibody DOTA-(Z_08699_)_3_ at different monomer:trimer molar ratios. HER3-expression in xenografts was imaged using [^57^Co]Co-NOTA-Z_08699_ and [^57^Co]Co-NOTA-Z_08699_: DOTA-(Z_08699_)_3_. Hepatic activity uptake of [^57^Co]Co-NOTA-Z_08699_: DOTA-(Z_08699_)_3_ decreased with increasing monomer:trimer molar ratio. The tumor activity uptake and tumor-to-liver ratios were the highest for the 1:3 ratio. SPECT/CT images confirmed the biodistribution data. Imaging of HER3 expression can be improved by co-injection of a radiolabeled monomeric affibody-based imaging probe together with a trivalent affibody.

## Introduction

Abnormal expression of cell-surface proteins such as receptor tyrosine kinases (RTK) is often associated with tumorigenesis or tumor(al) progression. Understanding the pattern and role of such aberrant expression enables a potential improvement in treating disseminated cancers with the development of targeting drugs. Molecular imaging of overexpressed proteins could be utilized for selecting patients who would benefit from a targeted therapy^1^.

The human epidermal growth factor receptor type 3 (HER3 or ErbB3), belonging to the type I RTK ErbB receptor family, has lately attracted attention as a molecular target for anticancer therapy^2–4^. HER3 and the other members of the ErbB receptor family have a general structure containing an extracellular binding domain (ECD), an intracellular tyrosine kinase domain, and an intracellular C-terminal tail. The intracellular tyrosine kinase domain of HER3 is inactive, but the receptor can form active heterodimers with other members of the ErbB receptor family. One of the most potent heterodimers in tumorigenesis is the HER2/HER3 pair which activates downstream signaling pathways, e.g. PI-3K/Akt and MAPK/MEK4^2,5^.

Although clinically approved antibody-based drugs against HER2 expressing breast cancer, trastuzumab (Herceptin) and pertuzumab (Perjeta), have shown to be effective, many researchers have reported resistance against therapy by upregulation of other receptors in the ErbB receptor family, particularly HER3^6,7^. The role of HER3-induced signaling in various tumors demonstrates its importance as a molecular target for therapy. Several monoclonal antibodies targeting HER3 are currently evaluated in the clinic^8,9^.

Molecular detection of HER3 expression for stratifying cancer patients who would respond to anti-HER3 treatment could be very beneficial. Currently, the predominant method for molecular profiling of tumors is biopsy. However, some issues are associated with this procedure, such as heterogeneous target expression in tumors and the invasiveness. Additionally, HER3 expression might change in response to applied therapy, meaning that the biopsy samples taken from the primary tumor will not be informative for metastases^10^. Radionuclide molecular imaging could help to overcome difficulties associated with the receptor expression heterogeneity and invasiveness. This would enable monitoring of receptor expression changes during treatment. The overexpression of HER3 in tumors is usually low, below 50,000 receptors/cell^11^. There is also endogenous expression of the protein in several organs, particularly the liver, which complicates imaging interpretation^11^. Therefore, imaging of HER3 expression is challenging and requires a probe with high affinity to HER3 (to increase uptake in lesions), rapid blood clearance and minimum uptake in healthy organs (to decrease background signal).

Affibody molecules are small (7 kDa) high-affinity scaffold proteins, which have demonstrated their targeting ability to several cancer-related receptors (e.g. EGFR, HER2 and IGF-1R) as well as their high potential as imaging agents^12^. Clinical studies using HER2-targeting affibody molecules have demonstrated their safety, specific targeting and high-contrast imaging^13,14^. Affibody molecules binding to HER3 with low picomolar affinity have recently been generated and described in several studies^15–21^. A HER3-targeting affibody molecule was also tested in a pre-clinical therapy study with multiple injections without any detected toxicity^22,23^. Different variants of the HER3-targeting affibody molecule were labeled with radioisotopes suitable for both single photon emission computed tomography (SPECT) and positron emission tomography (PET)^17,18,20,21^. It was also demonstrated that anti-HER3 affibody molecules could be used to monitor changes of the HER3-expression^18^. Importantly, HER3-targeting affibody molecules have similar affinities to HER3 and its murine counterpart, mErbB3, which makes mice a realistic model for targeting evaluation^23,24^.

Recently, we demonstrated that a radiocobalt labeled anti-HER3 affibody molecule had the most favorable biodistribution profile (among previously tested variants) with the lowest unspecific hepatic uptake accompanied by high and stable uptake in tumors^19^. We were interested in radiocobalt because ^55^Co (T_1/2_ = 17.5 h, 76% β^+^) enables PET imaging up to 24 h after injection. Cobalt-55 can be produced by cyclotrons and could therefore be available in the majority of PET facilities. We have also demonstrated that by optimization of the protein injected dose, activity uptake in liver but not in tumor could be suppressed^18^. Available data indicate that the hepatic uptake of anti-HER3 affibody molecules depends on two mechanisms, where one is HER3-mediated and the second is caused by unspecific interaction of the protein with hepatocytes^16–19^. For other affibody molecules (targeting HER1 and HER2), it was found that monomeric constructs had significantly higher tumor uptake than corresponding dimeric constructs^25,26^, most likely due to better extravasation^27^. Therefore, we hypothesized that the HER3-mediated hepatic uptake could be, to some extent, selectively saturated if a multimeric, non-labeled HER3-targeting affibody molecule would be co-injected with its radiolabeled monomeric form. A HER3-targeting trivalent affibody consisting of three linked anti-HER3 affibody molecules (Z_08699_)_3_ (~21 kDa) was thus generated.

In this study we investigated the influence of co-injection of the non-labeled trivalent anti-HER3 affibody molecule on biodistribution and tumor targeting of radiocobalt-labeled monomer ([^57^Co]Co-NOTA-Z_08699_, where NOTA is 1,4,7-triazacyclononane-N,N′,N′′-triacetic acid). For experimental convenience, the commercially available long-lived cobalt isotope ^57^Co (T_1/2_ = 271.8 d) was used as a surrogate of ^55^Co.

## Results

### (Z_08699_)_3_ characterization

**Figure 1:**
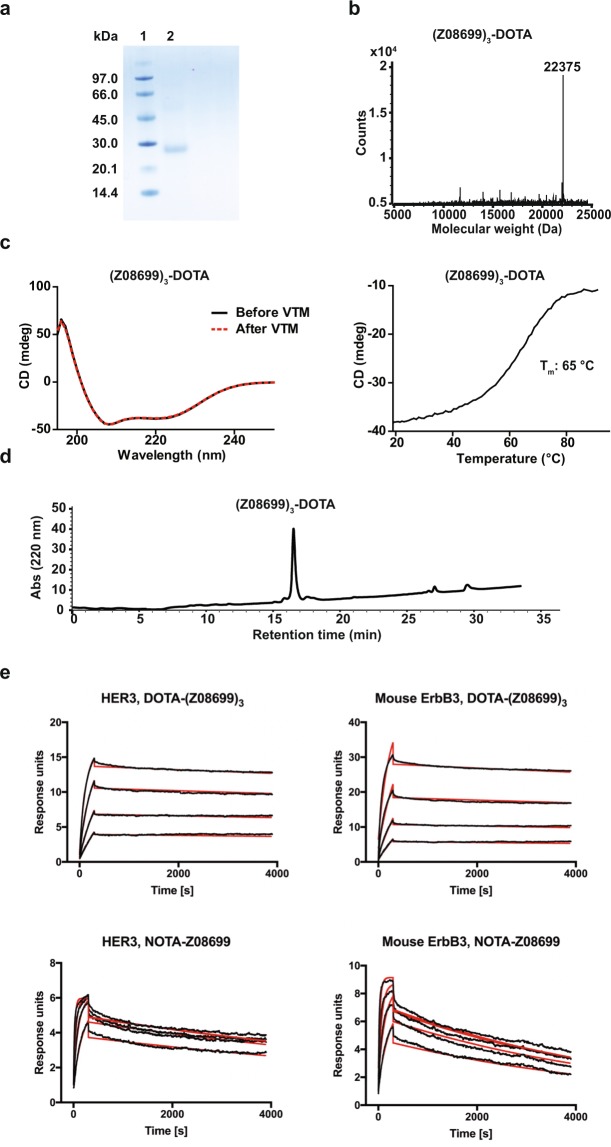
Characterization of DOTA-(Z_08699_)_3_. (**a**) SDS-PAGE of purified and DOTA-conjugated trivalent affibody. Lane 1: Mass ladder; Lane 2: DOTA-(Z_08699_)_3_. Full-length gel is presented in Supplementary Fig. 1. (**b**) ESI-MS mass determination for DOTA-conjugated trimer (estimated M_W_ 22375 Da). (**c**) Circular dichroism and variable temperature measurement before and after heating to 98 °C. (**d**) Chromatogram from analytical RP-HPLC measured using 220 nm absorbance. (**e**) Affinity determination of DOTA-(Z_08699_)_3_ and NOTA- Z_08699_ using multi-cycle kinetics on a Biacore T200 to immobilized HER3-mFc and mouse ErbB3. The injected concentrations were 20, 10, 5 and 2.5 nM for both conjugates. Experimental sensorgrams are shown in black and fitted data in red.

Analysis of the DOTA-conjugated (DOTA-1,4,7,10-tetraazacyclododecane-1,4,7,10-tetraacetic acid) and purified trivalent affibody using sodium dodecyl sulfate polyacrylamide gel electrophoresis (SDS-PAGE) showed pure recombinant protein of correct size (Fig. 1a). The molecular mass for DOTA-(Z_08699_)_3_ was determined to be 22375 Da, which is in perfect agreement with the calculated mass (22375 Da) (Fig. 1b). Circular dichroism spectroscopy during variable temperature measurement (VTM) demonstrated a melting temperature of 65 °C and excellent refolding capabilities of the construct (Fig. 1c). Analytical RP-HPLC measured using 220 nm absorbance demonstrated a 96% purity for DOTA-conjugated (Z_08699_)_3_ with retention time of 16.7 minutes (Fig. 1d). The kinetics of the binding reaction of DOTA-(Z_08699_)_3_ to recombinant HER3-mFc and mouse ErbB3 was determined using multi-cycle kinetics on a Biacore T200. For HER3, the association rate constant (k_a_) was 4.7 ± 0.8 × 10^5^ M^−1^s^−1^, and the dissociation rate constant (k_d_) was 5.3 ± 4.6 × 10^−5^ s^−1^, corresponding to an equilibrium dissociation constant (K_D_) of 108 ± 80 pM. For mouse ErbB3, k_a_ was 2.8 ± 0.9 × 10^5^ M^−1^s^−1^, and k_d_ was 8.4 ± 8.5 × 10^−5^ s^−1^, corresponding to a K_D_ of 263 ± 222 pM (Fig. 1e). The affinity of NOTA-Z_08699_ was included for comparison. For HER3, k_a_ was 3.9 ± 2.8 × 10^6^ M^−1^s^−1^, and k_d_ was 1.8 ± 1.0 × 10^−4^ s^−1^, corresponding to a K_D_ of 48 ± 9 pM. For mouse ErbB3, k_a_ was 1.7 ± 0.3 × 10^6^ M^−1^s^−1^, and k_d_ was 2.4 ± 0.6 × 10^−4^ s^−1^, corresponding to a K_D_ of 140 ± 11 pM (Fig. 1e).

### Labeling of DOTA-(Z_08699_)_3_ with ^111^In and *in vitro* characterization of [^111^In]In-DOTA-(Z_08699_)_3_

**Figure 2:**
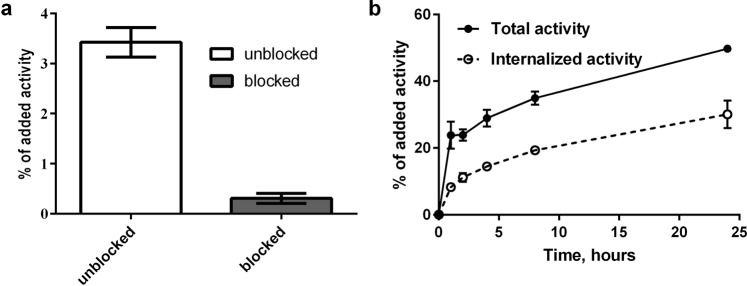
*In vitro* characterization of [^111^In]In-DOTA-(Z_08699_)_3_ using HER3 expressing cell line BxPC-3. (**a**) *In vitro* binding specificity test, cells were incubated 1 h at 4 °C with 0.1 nM of [^111^In]In-DOTA-(Z_08699_)_3_. The cell-associated activity was calculated as a percentage of the total added activity (mean values of three dishes ± SD). (**b**) Uptake and internalization of [^111^In]In-DOTA-(Z_08699_)_3_. Cells were continuously incubated with 0.1 nM solution of labeled conjugate at 37 °C. Data are presented as mean values from three samples ± SD. Error bars might not be seen because they are smaller than the symbols.

The trivalent affibody was labeled with ^111^In with a yield over 98% as determined by radioinstant thin layer chromatography and was used without further purification. The labeled trivalent affibody was stable under challenge with 500-fold molar excess of EDTA (ethylenediaminetetraacetic acid). Pre-saturation of receptors with non-labeled trivalent affibody demonstrated a significant decrease (p < 0.05) of the cell-associated activity when tested on HER3-expressing cells (Fig. 2a), confirming specificity of the trivalent affibody to HER3-receptors. The assay on cellular processing performed with BxPC-3 cells demonstrated a rapid binding and constant increase of cellular uptake of activity and internalized fraction over time (Fig. 2b). The internalized fraction reached 60% of total cell-associated activity at the 24 h time point, and the total cell-bound activity increased by 2-fold from 1 to 24 h during continuous incubation.

### *In vivo* studies using BxPC-3 tumor bearing mice

Direct comparison of biodistribution patterns 4 h after co-injection of molar equivalents of [^57^Co]Co-NOTA-Z_08699_ (0.5 µg) and [^111^In]In-DOTA-(Z_08699_)_3_ (1.5 µg) in tumor bearing mice is presented in Table 1. Radiolabeled trivalent affibody had significantly lower activity concentration in blood than radiolabeled monomer. Uptake in almost all studied normal organs and tissues was significantly higher for the trivalent affibody, except lungs (where it was equal to monomer) and kidneys (where monomer had significantly higher uptake). The most prominent was the difference in livers where uptake of trivalent affibody was 10-fold higher. However, tumor uptake was the same for monomer and trivalent affibody.

**Table 1:** Biodistribution of [^57^Co]Co-NOTA-Z_08699_ (0.5 µg) and [^111^In]In-DOTA-(Z_08699_)_3_ (1.5 µg) 4 h after co-injection in BxPC-3 xenografted mice (molar ratio 1:1).

	[^57^Co]Co-NOTA-Z_08699_	[^111^In]In-DOTA-(Z_08699_)_3_
Blood	0.50 ± 0.06^a^	0.20 ± 0.02
Salivary glands	1.0 ± 0.3	4.0 ± 0.5^a^
Lung	0.8 ± 0.1	0.8 ± 0.1
Liver	1.9 ± 0.1	21.1 ± 0.2^a^
Spleen	0.7 ± 0.2	5 ± 1^a^
Stomach	0.74 ± 0.06	3.0 ± 0.4^a^
Small intestine	2.3 ± 0.8	7 ± 1^a^
Kidney	283 ± 24^a^	131 ± 14
Tumor	1.3 ± 0.3	1.4 ± 0.4
Muscle	0.20 ± 0.05	0.40 ± 0.09^a^
Bone	0.36 ± 0.09	1.0 ± 0.2^a^
Gastrointestinal tract*	2.6 ± 0.3	6.7 ± 0.2^a^
Carcass*	6.8 ± 0.6	11 ± 1^a^

^a^value was significantly higher (paired, two-tailed t-test, p < 0.05).

The organ uptake values are expressed as a percentage of injected dose per gram of tissue weight (% ID/g). *Values for gastrointestinal tract with content and carcass are given as %ID per whole sample.

**Figure 3:**
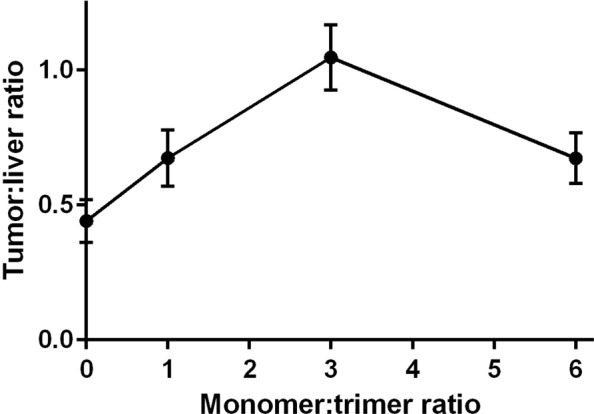
Tumor-to-liver ratio depending on monomer:trimer molar ratio 4 h after co-injection of [^57^Co]Co-NOTA-Z_08699_ (0.5 µg): DOTA-(Z_08699_)_3_ in BxPC-3 tumor bearing mice.

Additionally, biodistribution of [^57^Co]Co-NOTA-Z_08699_ (fixed labeled protein mass of 0.5 µg/animal) co-injected with unlabeled trivalent affibody DOTA-(Z_8699_)_3_ at monomer:trimer molar ratios of 1:0, 1:3 and 1:6 was studied 4 h post injection (pi) in BxPC-3 xenografted mice (Table 2). The activity concentration in blood after injection of 0.5 µg of monomer was significantly higher than for monomer co-injected with trivalent affibody for 1:1 and 1:6 ratios. The lowest concentration was found for a monomer:trimer molar ratio of 1:6. Generally, uptake in normal organs decreased with increased molar ratio. Hepatic activity uptake for sole monomer was significantly higher than for other tested combinations. Among the tested ratios, significantly lower hepatic uptake was found for ratio 1:6. However, tumor activity uptake was the highest for 1:3 ratio. The maximum tumor-to-liver ratio was found at 1:3 monomer:trimer ratio, 1.0 ± 0.1, which was significantly higher than for 1:0, 1:1 and 1:6 molar ratios (Fig. 3).

**Table 2:** Biodistribution of [^57^Co]Co-NOTA-Z_08699_ (0.5 µg): (Z_08699_)_3_ 4 h after being co-injected with different molar ratios in BxPC-3 tumor bearing mice.

Organs	[^57^Co]Co-NOTA-Z_08699_ (0.5 µg): DOTA-(Z_08699_)_3_ molar ratio			
	1:0	1:1 *	1:3	1:6
Blood	0.63 ± 0.04^a^	0.50 ± 0.06^e^	0.63 ± 0.06^f^	0.15 ± 0.01
Salivary glands	1.1 ± 0.2^c^	1.0 ± 0.3	1.0 ± 0.2	0.62 ± 0.03
Lung	0.9 ± 0.3	0.8 ± 0.1	1.0 ± 0.1^f^	0.6 ± 0.1
Liver	3.4 ± 0.2^a,b,c,^	1.9 ± 0.1^e^	1.81 ± 0.09^f^	1.43 ± 0.05
Spleen	0.66 ± 0.09	0.7 ± 0.2	1.0 ± 0.1	0.59 ± 0.09
Stomach	1.01 ± 0.06^c^	0.74 ± 0.06	0.88 ± 0.07^f^	0.48 ± 0.06
Small intestine	3.0 ± 0.2^b,c^	2.3 ± 0.8	1.31 ± 0.08^f^	0.69 ± 0.05
Kidney	276 ± 33	283 ± 24	346 ± 22	271 ± 29
Tumor	1.5 ± 0.3^c^	1.3 ± 0.3^d^	1.9 ± 0.1^f^	1.0 ± 0.1
Muscle	0.17 ± 0.03	0.20 ± 0.05	0.29 ± 0.02	0.19 ± 0.07
Bone	0.24 ± 0.04	0.36 ± 0.09	0.6 ± 0.1	0.27 ± 0.05
Gastrointestinal tract**	3.3 ± 0.9	2.6 ± 0.3	1.7 ± 0.2	1.5 ± 0.6
Carcass**	5.4 ± 0.9	6.8 ± 0.6	6 ± 4	4 ± 3

Data were assessed by one-way ANOVA with Bonferroni correction for multiple comparisons in order to determine significant differences (p < 0.05):

^a^value for 1:0 ratio was significantly higher than for 1:1 ratio;

^b^value for 1:0 ratio was significantly higher than for 1:3 ratio;

^c^value for 1:0 ratio was significantly higher than for 1:6 ratio;

^d^value for 1:1 ratio was significantly higher than for 1:3 ratio;

^e^value for 1:1 ratio was significantly higher than for 1:6 ratio;

^f^value for 1:3 ratio was significantly higher than for 1:6 ratio.

The organ uptake values are expressed as a percentage of injected dose per gram of tissue weight (% ID/g). *Data for 1:1 ratio are from Table 1 and are added for comparison. **Values for gastrointestinal tract with content and carcass are given as %ID per whole sample.

**Figure 4:**
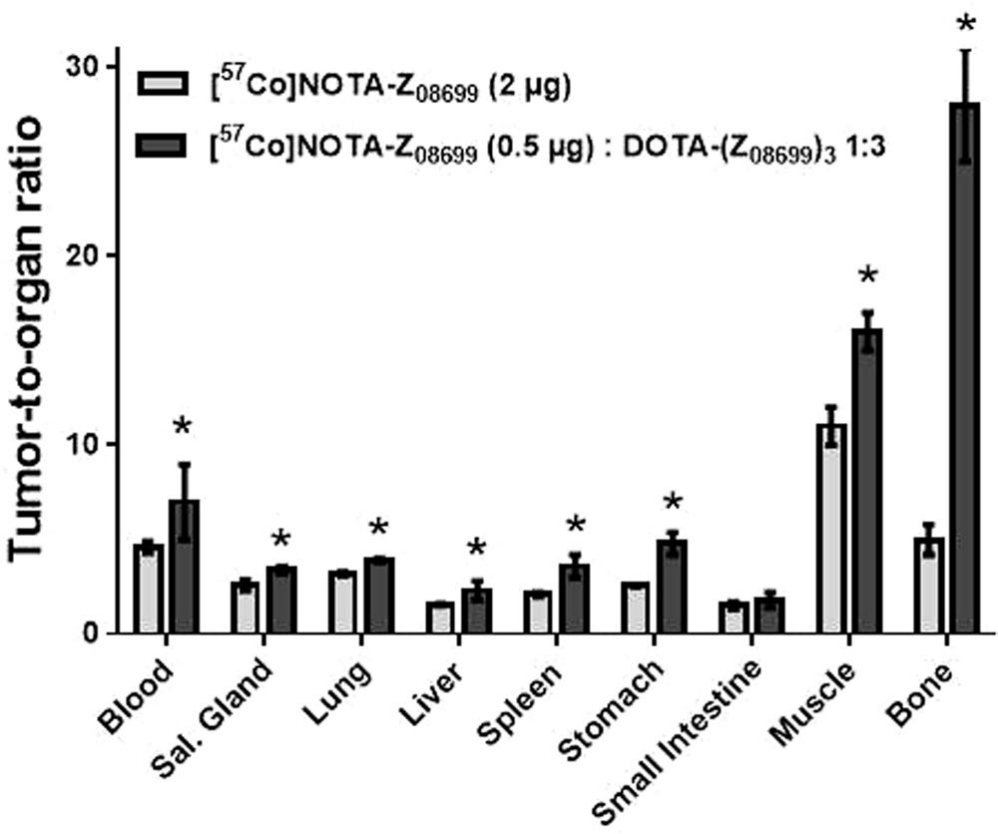
Comparison of tumor-to-organ ratios 24 h pi of [^57^Co]Co-NOTA-Z_08699_ (2 µg) or [^57^Co]Co-NOTA-Z_08699_ (0.5 µg): DOTA-(Z_08699_)_3_ (molar ratio 1:3) in BxPC-3 tumor bearing mice. Asterisks mark organs with significantly different values.

The biodistribution of [^57^Co]Co-NOTA-Z_08699_ (0.5 µg) co-injected with DOTA-(Z_08699_)_3_ in molar ratio 1:3 was compared with biodistribution of [^57^Co]Co-NOTA-Z_08699_ (2 µg) 24 h pi (the best performing dose and time point among previously published^19^). Data for this experiment are presented in Table 3 and Fig. 4. Data for injection of 2 µg of [^57^Co]Co-NOTA-Z_08699_ were in good agreement with previously published^19^. Head-to-head comparison 24 h pi of [^57^Co]Co-NOTA-Z_08699_ and [^57^Co]Co-NOTA-Z_08699_: DOTA-(Z_08699_)_3_ (1:3) demonstrated that tumor activity uptake was significantly higher in the case of co-injection of trivalent affibody. Other tested organs and tissue did not differ significantly in activity uptake (except spleen that had lower activity uptake in the group that was co-injected with trivalent affibody). Tumor-to-organ ratios were significantly higher (except tumor-to-small intestine) for the group that was co-injected with trivalent affibody (Fig. 4). Tumor-to-liver ratio increased with time and was 2.3 ± 0.5 24 h pi.

**Table 3:** Biodistribution of [^57^Co]Co-NOTA-Z_08699_ (2 µg) and [^57^Co]Co-NOTA-Z_08699_ (0.5 µg) co-injected with DOTA-(Z_08699_)_3_ (molar ratio 1:3) 24 h pi in BxPC-3 tumor bearing mice.

Organs	[^57^Co]Co-NOTA-Z_08699_ (2 µg)	[^57^Co]Co-NOTA-Z_08699_ (0.5 µg): (Z_08699_)_3_ (1:3)
Blood	0.47 ± 0.03	0.39 ± 0.07
Salivary Gland	0.84 ± 0.05	0.81 ± 0.10
Lung	0.68 ± 0.03	0.9 ± 0.4
Liver	1.4 ± 0.1	1.2 ± 0.1
Spleen	1.04 ± 0.04^a^	0.78 ± 0.08
Stomach	0.76 ± 0.09	0.64 ± 0.07
Small Intestine	1.4 ± 0.1	1.6 ± 0.5
Kidney	179 ± 24	172 ± 22
Tumor	2.1 ± 0.1	2.8 ± 0.4^a^
Muscle	0.19 ± 0.03	0.17 ± 0.03
Bone	0.3 ± 0.1	0.15 ± 0.06
Gastrointestinal tract*	1.4 ± 0.9	2.3 ± 0.7
Carcass*	5.8 ± 0.4	6.2 ± 0.5

^a^value was significantly higher (un-paired, two-tailed t-test, p < 0.05).

The organ uptake values are expressed as a percentage of injected dose per gram of tissue weight (% ID/g). *Values for gastrointestinal tract with content and carcass are given as %ID per whole sample.

### Imaging studies

**Figure 5:**
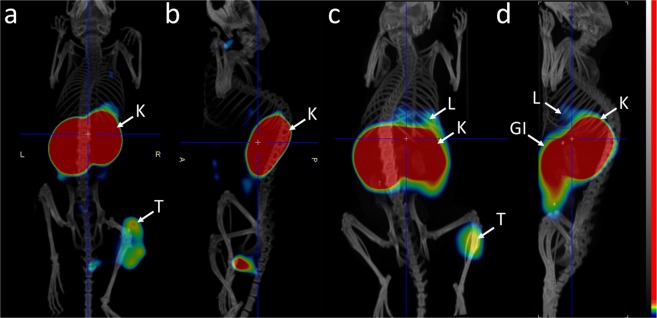
microSPECT/CT images of mice bearing BxPC-3 xenografts at 24 h pi. Coronal and sagittal projections of animals injected with (**a**,**b**) 0.5 µg of [^57^Co]Co-NOTA-Z_08699_: (Z_08699_)_3_ (molar ratio 1:3) or (**c**,**d**) 2 µg of [^57^Co]Co-NOTA-Z_08699_. Arrows indicate tumor (T), liver (L), and kidneys (K).

SPECT/CT imaging of HER3 expression in BxPC-3 xenografts confirmed the results of *ex vivo* measurements (Fig. 5). Tumors were clearly visualized 24 h pi of either [^57^Co]Co-NOTA-Z_08699_ (2 µg) or [^57^Co]Co-NOTA-Z_08699_ (0.5 µg): DOTA-(Z_08699_)_3_ (molar ratio 1:3). However, liver activity uptake was appreciably lower after co-injection with trivalent affibody.

## Discussion

Endogenous expression of a molecular target in healthy tissue may be an issue in imaging procedures. The target-specific uptake of imaging probes in normal organs reduces contrast of tumor imaging. This problem is relatively easy to solve when target expression in normal tissue is appreciably lower than in tumors. It has been demonstrated for somatostatin analogues^28^, scaffold proteins (affibody molecules^14^), and monoclonal antibodies (trastuzumab^29^) that the use of an optimal injected protein dose may saturate target receptors in normal tissues but not in tumors and improve the imaging contrast. However, HER3 is a challenging target due to relatively low expression in tumors, and more sophisticated approaches are hence required. The aim of this project was to test the hypothesis whether or not the imaging contrast of HER3 expression using affibody molecules can be improved by a combination of an optimal molecular design of the tracer (combination of NOTA and radiocobalt as a label^19^) and preferable saturation of HER3/mErbB3 in liver by co-injection of a multimeric HER3-specific affibody molecule. It was expected that the bulky trivalent affibody would have lower extravasation and tumor penetration rate compared with smaller monomer, which should prevent target saturation in tumors. The use of dimeric form of single-domain antibodies (sdAbs, nanobody, 12–15 kDa) for selective saturation of targets in the liver but not in tumor resulted in appreciably higher contrast in imaging of tumor-associated macrophages^30^. A different approach that could be of great interest to investigate further in preclinical models is the possibility to saturate the hepatic uptake by first injecting unlabeled monomeric HER3-targeting affibody molecule. Following this saturation, a second injection with radiolabeled monomer could be done and the tumor uptake could be further increased, leading to higher feasibility for future clinical imaging.

Slower dissociation of a trivalent affibody might be important for preferable binding to receptors in liver, where well-fenestrated vasculature would provide similar access for both monomer and trivalent affibody to receptor-expressing hepatocytes. One could expect slower dissociation due to avidity effect similar to anti-HER1 and anti-HER2 affibody molecules^31,32^ or anti-CD206 and anti-CD20 sdAbs^30,33^. Indeed, the dissociation rate of the trivalent affibody was three-fold slower than for the monomer.

A DOTA chelator was incorporated into (Z_08699_)_3_ to permit radiolabeling and simplify cellular processing and biodistribution studies. Indium-111 was chosen as a label for DOTA-(Z_08699_)_3_ in the dual-label animal studies for comparison with [^57^Co]Co-NOTA-Z_08699_. This strategy allows for simultaneous analysis of the distribution for both probes in the same animals. The binding specificity of DOTA-(Z_08699_)_3_ to HER3-expressing cells *in vitro* was preserved after labeling with indium-111 (Fig. 2a). The cellular processing of radiolabeled trivalent affibody demonstrated an internalization pattern similar to anti-HER3 monomer^16^, i.e. relatively rapid internalization (50% at 4 h) (Fig. 2b). The *in vitro* characterization of radiolabeled trivalent affibody was performed to corroborate that its targeting and biological properties (target specificity and internalisation pattern) were preserved after labeling that allowed us to perform dual isotope study *in vivo*. However, internalization of HER3/mErbB3 in liver after binding of DOTA-(Z_08699_)_3_ should be favorable for preventing hepatic uptake of radiolabeled monomer.

The direct comparison of [^111^In]In-DOTA-(Z_08699_)_3_ to [^57^Co]Co-NOTA-Z_08699_ demonstrated (Table 1) that the trimeric construct had significantly higher uptake in mErbB3-expressing organs, such as salivary gland, liver, spleen and small intestines. At the same time, activity concentration in blood was significantly lower for trivalent affibody than for the monomer that could be explained by sequestering of the conjugate by normal organs. This experiment confirmed that hepatic uptake of the trivalent affibody is by one order of magnitude higher than the hepatic uptake of the monomer. Further experiments (Table 2, Fig. 3) demonstrated that co-injection of DOTA-(Z_08699_)_3_ and [^57^Co]Co-NOTA-Z_08699_ at a 1:3 molar ratio provided the best tumor-to-liver ratio for the radiolabeled affibody molecule. Obviously, re-optimization would be required for clinical application. A previous study has demonstrated that tumor-to-liver ratio increased with time because retention of activity was better in the tumor in comparison to the liver^19^. Blocking hepatic uptake with DOTA-(Z_08699_)_3_ resulted in a tumor-to-liver ratio of 2.3 ± 0.5 at 24 h pi, which is the best value for HER3-targeting affibody molecules obtained in preclinical studies. We would like to point out that the increased tumor-to-liver ratio is a qualitative, rather than quantitative change, providing us with an improved imaging contrast. A higher tumor-to-liver ratio for co-injection of DOTA-(Z_08699_)_3_ could also be observed in microSPECT images (Fig. 5). In combination with ^18^F-FDG PET for initial lesion detection, this should enable visualization of HER3 expression in hepatic metastases, which are common in many types of cancer. Other tumor-to-organ ratios were also significantly higher at 24 h with the co-injection of the trimer compared to the optimal injected protein dose of the monomer (Fig. 4). The use of ^55^Co as a label would enable imaging 24 h after injection.

In conclusion, imaging of HER3 expression in tumors can be improved by co-injection of a radiolabeled monomeric affibody-based imaging probe together with an trivalent affibody.

## Material and Methods

### Materials

The cell line BxPC-3 (pancreatic carcinoma) used during *in vitro* and *in vivo* experiments was purchased from American Type Tissue Culture Collection (ATCC via LGC Promochem, Borås, Sweden). Production and characterization of the monomeric affibody molecule NOTA-Z_08699_ was previously published^17^. The activity content in samples was measured using automated gamma-counter with 3-inch NaI(Tl) detector (1480 WIZARD, Wallac Oy). Data were assessed by an unpaired, two-tailed t-test or by one-way ANOVA with Bonferroni correction for multiple comparisons using GraphPad Prism (version 6 for Windows GraphPad Software) in order to determine significant differences (p < 0.05). Obtained values are presented as average with standard deviation if not stated otherwise.

### Construction and production of (Z_08699_)_3_

Trimeric affibody molecule containing three monomeric Z_08699_ affibody molecules interlinked by a VE-linker capped by an N-terminal HE-tag and a unique C-terminal cysteine was constructed using conventional restriction enzyme cloning. The trimeric affibody is denoted (Z_08699_)_3_. The construct was cloned into pET26b+ vector (Novagen) and Sanger sequencing was used to confirm the correct insert. The construct was transformed into *E. coli* BL-21 (DE3) and produced by growing bacteria in tryptic soy broth containing kanamycin. Protein production was induced when OD_600nm_ reached 0.5 by addition of 1 mM of Isopropyl β-D-1-thiogalactopyranoside (IPTG), and the culture was incubated at 25 °C overnight. Bacteria were lysed using Aminco french press (Laurier Research Instrumentation).

### Purification, conjugation and characterization of DOTA-(Z_08699_)_3_

The sample was purified by heat treatment at 70 °C for 10 minutes followed by centrifugation at 30,000 g at 4 °C for 10 minutes to remove precipitated host proteins. Following this, the C-terminal cysteine was conjugated with maleimide-DOTA (Chematech, Dijon, France) after reduction of the thiols followed by an addition of 3-fold molar excess of chelator in ammonium acetate buffer, pH 5.5. The mixture was incubated at 40 °C for 40 minutes. The conjugate was purified using RP-HPLC with a preparative Zorbax C18 column and a gradient 20–40% of 0.1% trifluoroacetic acid in acetonitrile for 35 min at a flow rate of 3 ml/min on a 1200 series RP-HPLC (Agilent Technologies). Analysis of purity was measured by RP-HPLC (Zorbax 300SB-C18; Agilent Technologies, Inc., Palo Alto, CA, USA) using a gradient 20–40% of 0.1% trifluoroacetic acid in acetonitrile for 36 min at a flow rate of 1 ml/min. Affinity against recombinant HER3-mFc and mouse ErbB3 was analyzed in duplicates using multi-cycle kinetics on a Biacore T200 (GE Healthcare, Uppsala, Sweden). For comparison, NOTA-Z_08699_ was analyzed in the same run. The injected concentrations were 20, 10, 5 and 2.5 nM for both conjugates. Mass was determined using ESI-MS, 6520 Accurate-Mass Q-TOF LC/MS (Agilent Technologies) and the melting temperature was measured using circular dichroism spectroscopy (Jasco Inc., Easton, MD, USA).

### Labeling

Radiolabeling of DOTA-(Z_08699_)_3_ with ^111^In was performed similarly to the method previously described^17^, and labeling of NOTA-Z_08699_ (further denoted Z_08699_) with ^57^Co was performed as reported previously^19^. To determine the radiochemical yield and radiochemical purity, the labeled proteins were analyzed by radio instant thin-layer chromatography (radio-ITLC, 150–771 DARK GREEN, Tec-Control Chromatography strips from Biodex Medical Systems, New York, USA) eluted with 0.2 M citric acid, pH 2.0. For stability validation, samples of the radiolabeled protein were diluted with 500-fold molar excess of ethylenediaminetetraacetic acid (EDTA) in phosphate-buffered saline (PBS), control samples were diluted with equal amounts of PBS, and they were further incubated at room temperature for 1 h and analyzed by radio-ITLC.

### *In vitro* specificity test and investigation of cellular processing for [^111^In]In-DOTA-(Z_08699_)_3_

The binding specificity of [^111^In]In-DOTA-(Z_08699_)_3_ to HER3-expressing cells and its cellular processing were studied in BxPC3 cells. The *in vitro* specificity test was performed in triplicates following the method described earlier^19^. Cells were incubated for 1 h at 4 °C with solutions of ^111^In-labeled trivalent affibody (0.1 nM) with or without non-labeled trivalent affibody (100 nM) and the values of cell-associated activity were compared. For the cellular processing experiment, cells were continuously incubated at 37 °C with 0.1 nM solution of ^111^In-labeled trivalent affibody and the internalized and membrane bound activity were measured at pre-determined time points as described earlier^19^.

### *In vivo* studies

All animal experiments were planned and performed in accordance with Swedish national legislation on laboratory animals’ protection and were approved by the local Ethics Committee for Animal Research in Uppsala. Groups of four mice were used per data point. BALB/C nu/nu mice bearing BxPC-3 xenografts were used to study the targeting properties and biodistribution of radiolabeled affibody conjugates. Cells (5 × 10^6^ cells/mouse) were implanted subcutaneously 3 weeks before the experiment. At the time of the experiment the mice weight was 18 ± 1 g and the tumor weight was 0.31 ± 10 g.

One group of mice was i.v. injected with 0.5 µg of [^57^Co]Co-NOTA-Z_08699_ and 1.5 µg of [^111^In]In-DOTA-(Z_08699_)_3_ (molar ratio 1:1)_._ In another experiment [^57^Co]Co-NOTA-Z_08699_ (fixed labeled protein mass of 0.5 µg/animal) was co-injected with unlabeled trivalent affibody DOTA-(Z_8699_)_3_ at monomer:trimer molar ratios of 1:0, 1:3 and 1:6. Mice were sacrificed at 4 h post injection (pi), by heart puncture with a heparinized syringe under anesthesia. Samples of blood, organs and tumors were collected and uptake of activity in tissues was measured. Tissue uptake was calculated as the percentage of the injected activity per gram tissue weight (%ID/g). Activity uptake in the carcass and the gastrointestinal tract with content was calculated as %ID per whole sample. In additional experiment biodistribution of 2 μg of [^57^Co]Co-NOTA-Z_08699_ in BxPC-3 xenografted mice was compared with 0.5 μg of [^57^Co]Co-NOTA-Z_08699_ co-injected with 4.5 μg of DOTA-(Z_08699_)_3_ (molar ratio 1:3) at 24 h pi.

### Imaging

Mice bearing BxPC-3 xenografts were iv injected with 0.5 μg of [^57^Co]Co-NOTA-Z_08699_ and 4.5 μg of DOTA-(Z_08699_)_3_ (molar ratio 1:3) or with 2 µg [^57^Co]Co-NOTA-Z_08699_. The mice were euthanized and imaged at 24 h pi using Triumph™ Trimodality System (Gamma Medica), an integrated microSPECT/CT platform. The computed tomography (CT) acquisition: FOV, 80 mm; magnification,1.48; one projection, 512 frames. SPECT acquisition: FOV, 80 mm; 5 pinhole collimators; 64 projections. CT raw files were reconstructed by filter back projection (FBP). SPECT raw data was reconstructed by the FLEX™ SPECT software, which uses an ordered subset expectation maximization (OSEM) iterative reconstruction algorithm. SPECT and CT data were fused and analyzed using PMOD v3.508 (PMOD Technologies Ltd., Zurich, Switzerland). CT acquisition: CT-energy peak of 50 keV, 670 μA, 480 projections, 2.29 min acquisition time. SPECT acquisition: energy window, 109.89–134.31 keV, 110 projection, matrix of 256 × 256. CT raw files were reconstructed in real time using Nucline 2.03 Software (Mediso Medical Imaging Systems). SPECT raw data were reconstructed using Tera-Tomo™ 3D SPECT reconstruction technology.

## Supplementary information

Suppl.Figure 1
