# Supplementary material for: Improved contrast of affibody-mediated imaging of HER3 expression in mouse xenograft model through co-injection of a trivalent affibody for in vivo blocking of hepatic uptake
Source: Sci Rep. 2019 May 1;9:6779. doi: 10.1038/s41598-019-43145-2 (PMC6494909; doi:10.1038/s41598-019-43145-2)
Supplement: Supplementary file 1 — Suppl.Figure 1 [file 41598_2019_43145_MOESM1_ESM.pdf]

Supplementary Data File

Improved contrast of affibody-mediated imaging of HER3 expression in mouse xenograft model through co-injection of a trivalent affibody for in vivo blocking of hepatic uptake

Maria Rosestedt*[1], Ken G. Andersson*[2], Sara S. Rinne*[1], Charles Dahlsson Leitao[2], Bogdan Mitran[1], Anzhelika Vorobyeva3, Stefan Ståhl[2], John Löfblom[2], Vladimir Tolmachev[3], Anna Orlova[1,4]
[1]Department of Medicinal Chemistry, Uppsala University, Sweden;
[2]Division of Protein Technology, KTH – Royal Institute of Technology, Stockholm, Sweden;
[3]Department of Immunology, Genetics and Pathology, Uppsala University, Sweden
[4]Science for Life Laboratory, Uppsala University, Sweden

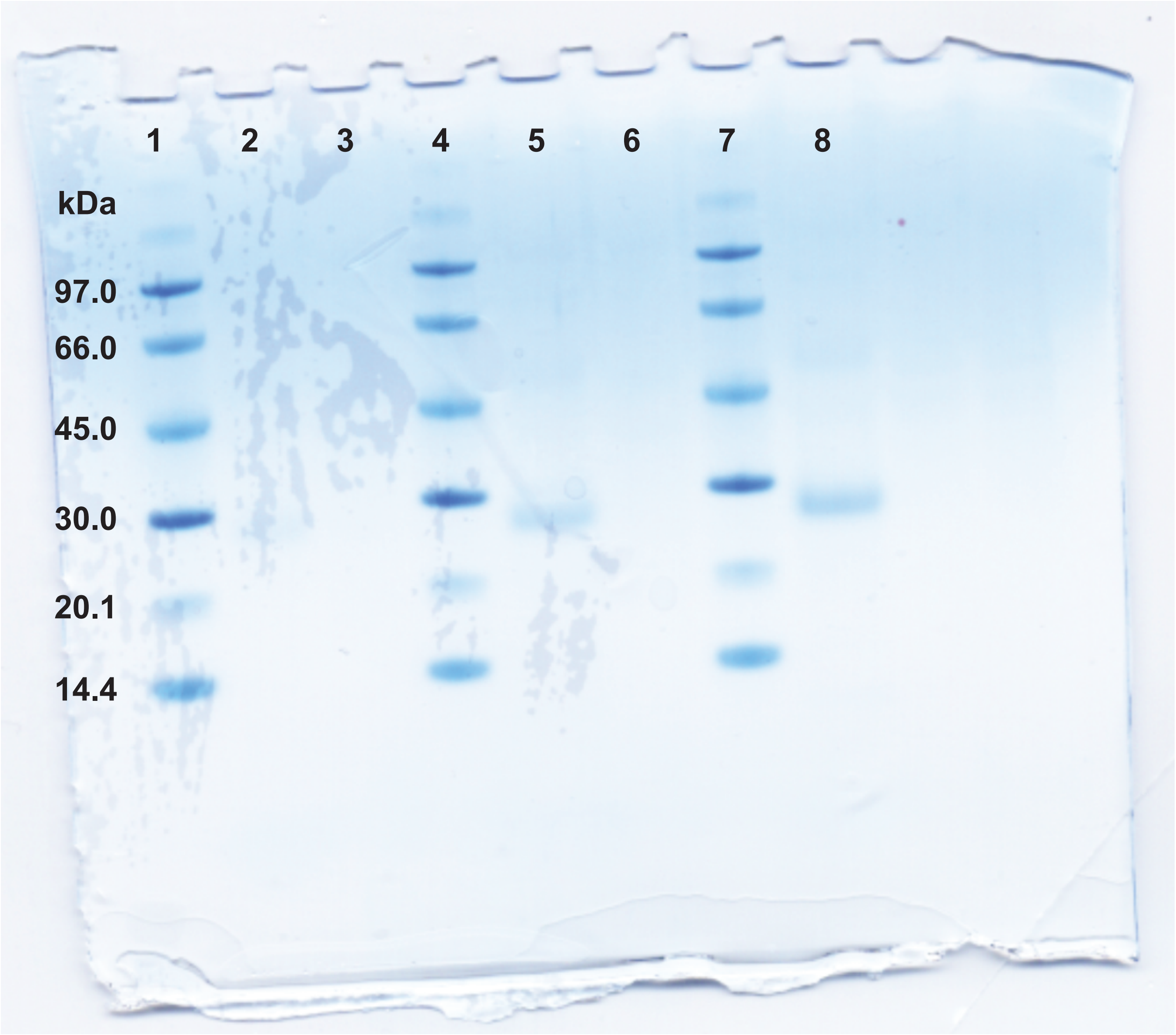

Supplementary Figure 1. Characterization of DOTA-$(Z_{08699})_3$. SDS-PAGE of purified and DOTA-conjugated trivalent affibody. Lane 1: Mass ladder; Lane 2: DOTA-$(Z_{08699})_3$, 5-fold dilution; Lane 3: Empty; Lane 4: Mass ladder; Lane 5: DOTA-$(Z_{08699})_3$, 2-fold dilution; Lane 6: Empty; Lane 7: Mass ladder; Lane 8: DOTA-$(Z_{08699})_3$, no dilution.
